# Gastric metastasis from sphenoid sinus melanoma: A case report

**DOI:** 10.3892/ol.2014.2745

**Published:** 2014-11-28

**Authors:** LIANJUN ZHAO, JING YAN, LI LI, JIA WEI, LIN LI, XIAOPING QIAN, BAORUI LIU, ZHENGYUN ZOU

**Affiliations:** 1 Comprehensive Cancer Center of Nanjing Drum Tower Hospital, Clinical College of Nanjing Medical University, Nanjing, Jiangsu 210008, P.R. China; 2 Comprehensive Cancer Center of Nanjing Drum Tower Hospital, Medical School and Clinical Cancer Institute of Nanjing University, Nanjing, Jiangsu 210008, P.R. China

**Keywords:** sphenoid sinus melanoma, gastric metastasis, gastrectomy

## Abstract

Clinical reports of primary sphenoid sinus melanoma and isolated gastric metastatic melanoma are rare. Thus, to the best of our knowledge, the present study reports the first case of isolated gastric metastasis from a sphenoid sinus melanoma. The aim of the present study was to discuss the clinicopathological and radiographic characteristics, the treatment strategy and the prognosis of sphenoid sinus metastatic malignant melanoma of the stomach. Although almost 60% of patients who succumb to melanoma exhibit gastrointestinal metastases at autopsy, antemortem diagnosis is uncommon; this is predominantly due to gastric metastatic melanoma presenting with non-specific symptoms similar to other common gastrointestinal diseases. Gastrectomy may prolong overall survival and improve the quality of life for gastric metastatic melanoma patients, and the present case emphasizes the importance of palliative surgery in such cases.

## Introduction

Malignant melanoma is an aggressive, therapy-resistant disease. Since the 1960’s, the global incidence of malignant melanoma has risen by 3–8% annually (1–2). Biopsy of the primary lesions and sentinel lymph nodes must be performed for optimal diagnosis and staging (1). Surgery is the main treatment used for melanoma, however, chemotherapy, radiotherapy and immunotherapy may also present promising therapeutic strategies (3). Malignant melanoma originating from the sphenoid sinus is rare and only eight cases have previously been reported (4–11). However, no gastric metastasis had been reported among the eight cases, as isolated gastric metastasis from malignant melanoma is also rare. To the best of our knowledge, the present study reports the first case of gastric metastasis from sphenoid sinus melanoma. Written informed consent was obtained from the patient’s family.

## Case report

**Figure 1:**
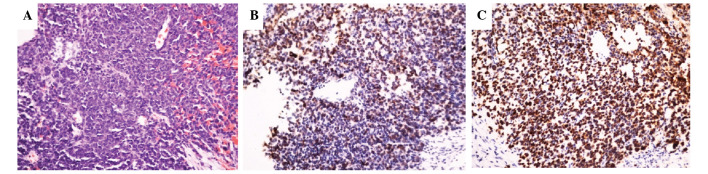
Histopathological biopsies obtained following a subtotal tumorectomy of left sphenoid sinus tumor using a nasal endoscope. The pathological diagnosis was of a malignant melanoma. (A) Hematoxylin-eosin staining showing large polygonal cells with large and deeply stained nuclei and positive staining for (B) HMB45 and (C) S100 (magnification, ×200).

**Figure 2:**
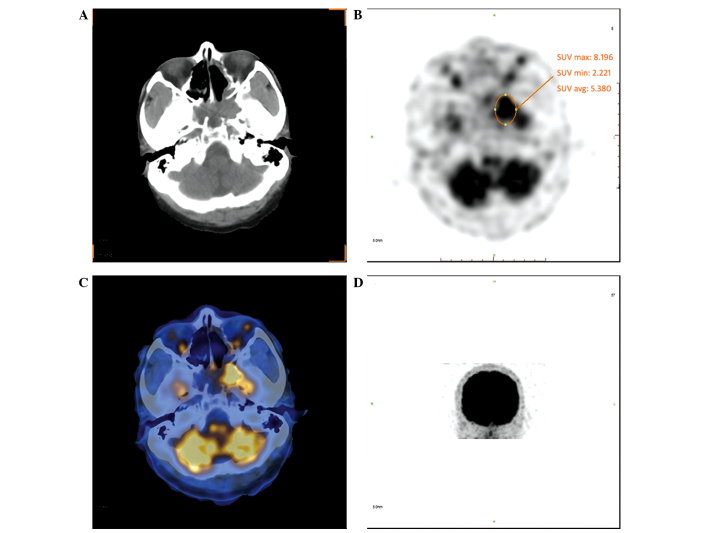
Post-operative fluorodeoxyglucose PET/CT indicating that the sphenoid sinus mass had not been completely removed during surgery (as shown by the circle).(A) CT, (B) attenuation corrected PET, (C) fusion PET/CT and (D) non-attenuation corrected PET images. SUV, standard uptake value; PET, positron emission tomography; CT, computed tomography.

**Figure 3:**
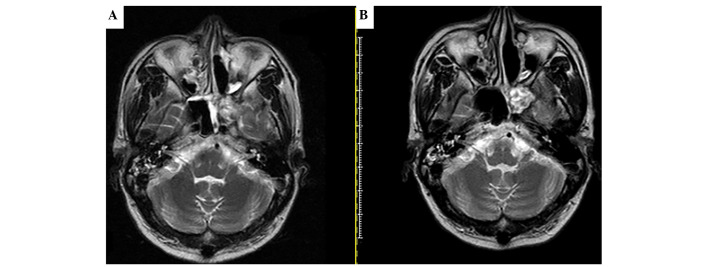
(A) MRI scan following two cycles of comprehensive chemotherapy and immunotherapy treatment. (B) MRI scan following four cycles of comprehensive chemotherapy and immunotherapy treatment, which determined that the patient had achieved a stable disease state, according to the Response Evaluation Criteria in Solid Tumors guidelines. MRI, magnetic resonance imaging.

A 57-year-old male was admitted to the Drum Tower Hospital (Nanjing, China) with the initial symptoms of nasal congestion and epistaxis. Magnetic resonance imaging (MRI) of the brain and paranasal sinuses indicated a sphenoid sinus mass. The mass filled the sphenoid sinus, and extended into the left ethmoidal sinus and the left optic foramen. The patient received a subtotal tumorectomy of the left sphenoid sinus tumor using a nasal endoscope. The pathological diagnosis was of a malignant melanoma (Fig. 1). The patient had no history of previously diagnosed cutaneous or mucosal melanomas at other sites. Additional conservative management with radiotherapy was implemented, as a post-operative fluorodeoxyglucose positron emission tomography/computed tomography (PET/CT) demonstrated that the sphenoid sinus mass had not been completely removed during surgery (Fig. 2). The total radiation dose was 60 Gy (2 Gy/30 fractions). Following radiation therapy, genetic testing of the peripheral blood was performed and it was identified that the patient was positive for O^6^-methylguanine-DNA methyltransferase (MGMT) methylation. Therefore, it was proposed that a combination of fotemustine (100 mg/m^2^, days 1 and 15) and gemcitabine hydrochloride (1,000 mg, day 1) chemotherapy, as well as cascade-primed immune cell immunotherapy (five times a week) should be administered to control the disease. Following four cycles of comprehensive treatment, which lasted for four months, the patient underwent a second MRI scan of the brain, and it was determined that the patient had achieved a stable disease state, according to the Response Evaluation Criteria in Solid Tumors guidelines (12) (Fig. 3). Subsequently, the patient received subcutaneous injections of 3×10^6^ units of interferon α-1b, three times a week.

**Figure 4:**
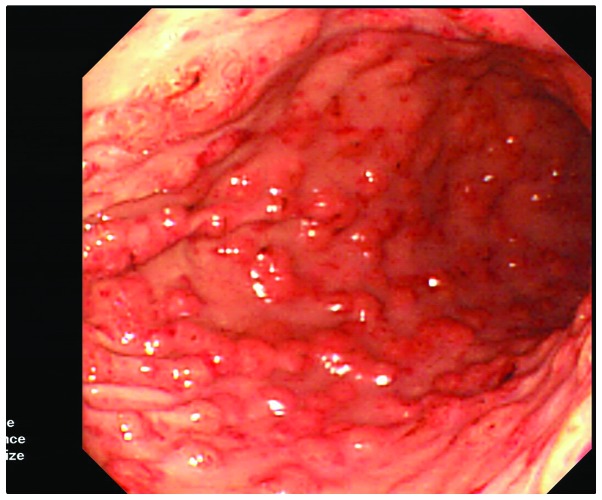
Identification of numerous small erosions and hyperplastic eminence lesions during diagnostic gastroscopy.

**Figure 5:**
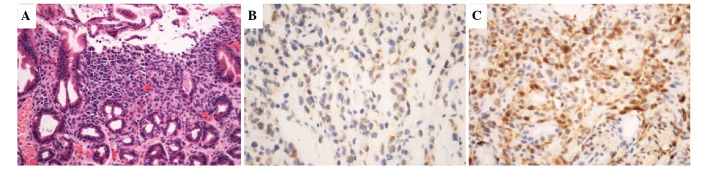
(A) Histopathological analysis of an incisional biopsy taken during gastroscopy revealing a malignant tumor (magnification, ×200). Hematoxylin and eosin staining showing (A) large polygonal cells with large and deeply stained nuclei and and positive staining for (B) HMB45 and (C) S100, which resulted in a diagnosis of metastatic malignant melanoma (stain, hematoxylin and eosin; magnification, ×400).

The patient returned to the Drum Tower Hospital six months later with the predominant complaint of diarrhea. During the diagnostic gastroscopy, numerous small erosions and hyperplastic eminence lesions were discovered, and an incisional biopsy was performed (Fig. 4). Histopathological analysis revealed a malignant tumor, and immunohistochemical staining determined that the tumor was S100- and HMB45-positive, which resulted in a diagnosis of metastatic malignant melanoma (Fig. 5). Abdominal ultrasound, head and neck MRI, and otolaryngological endoscopy were performed; these procedures indicated no other organ involvement. Gastrectomy to control the disease progression and relieve the symptoms was strongly recommended, however, the patient refused and succumbed two months later.

## Discussion

The incidence of malignant melanoma is increasing worldwide. Malignant melanoma is divided into cutaneous malignant melanoma and mucosal malignant melanoma according to its location. Mucosal malignant melanoma counts for 1–2% of malignant melanoma cases in the Caucasian population, but 22.6% in the ethnic Chinese population (13). Furthermore, primary malignant melanoma of the paranasal sinuses is rare, with a study by Osguthorpe (14) identifying that paranasal sinus neoplasms account for just 2–3% of head and neck cancers. In a clinicopathological study of 277 patients with malignant tumors of the nasal cavity and paranasal sinuses, malignant melanoma accounted for only 9% of cases (15). Sphenoid sinus malignant melanoma in particular are extremely rare, and only eight cases have previously been reported in the literature (4–11). Of these eight cases, no gastric metastasis was noted, thus, to the best of our knowledge, the present study is the first report of isolated gastric metastasis from a sphenoid sinus malignant melanoma.

Malignant melanoma patients typically exhibit unique symptoms. According to the previously reported cases, headaches, epistaxis and visual symptoms were common initial symptoms. Generally, however, the studied gastric metastatic malignant melanoma patients exhibited few typical clinical manifestations, instead presenting with fairly non-specific symptoms similar to other common gastrointestinal diseases, such as stomachaches, diarrhea, weakness, nausea, constipation and anemia. In the present case, nasal congestion and epistaxis were the initial symptoms, followed by diarrhea upon the development of gastric metastasis. Considering the innocuous gastrointestinal symptoms, metastatic melanoma should be a diagnostic candidate in any patients with a history of malignant melanoma (16).

The metastasis of malignant melanoma generally occurs via lymph node spread, although hematogenous metastasis may occur. In a previous study of 216 autopsy cases, the most common metastatic locations were the lymph nodes (73.6%), and lungs (71.3%), followed by the liver (58.3%), brain (54.6%), bones (48.6%) and adrenal glands (46.8%) (17). Clinical reports of gastric metastatic melanoma are rare, with the rate of confirmed abdominal cavity melanoma metastasis reported as only 2%. However, a previous study has shown that in 60% of patients who succumb to malignant melanoma, tissue sections reveal metastases to the gastrointestinal tract (18). In another autopsy study of patients who succumbed to metastatic cutaneous melanoma, gastric metastasis was reported in ≤27% of cases (19). Thus, gastric metastatic melanoma is not rare, but its non-specific clinical manifestations result in a low diagnostic accuracy.

The imaging manifestations of sphenoid sinus melanoma vary between cases. CT scans are a good way of identifying subtle bone erosion in the sphenoid sinus, while MRI is more effective in determining the extent of the tumor, vascular encasement, and perineural or subtle intracranial spread (11). The melanin content of a tumor determines its appearance on MRI; for example, melanotic melanoma demonstrates a hyperintense signal on T1-weighted imaging and a hypointense signal on T2-weighted imaging, whereas amelanotic melanoma demonstrates a hypointense or isointense signal on T1-weighted imaging and a hyperintense or isointense signal on T2-weighted imaging (9). Furthermore, distant metastasis may occur, thus, PET scanning is emerging as the preferred diagnostic modality for whole body metastatic evaluation. Gastric metastasis, however, may occasionally only be identifiable under gastroscopy.

Due to the rare nature of malignant melanoma of the sphenoidal sinus, the guidelines for sinonasal melanoma are used to devise a treatment strategy (10). Malignant melanomas are highly resistant to chemotherapy. The first-line chemotherapeutic agents that are used are the methylating agents dacarbazine and temozolomide, and the chloroethylating agents bis-chloroethylnitrosourea and fotemustine. In the present case, the use of fotemustine was proposed according to the peripheral blood genetic testing result, in which the patient was MGMT methylation-positive (20).

In a retrospective study of 124 gastrointestinal metastatic melanoma patients, 69 (55.6%) underwent surgical exploration of the abdomen. Of the 69 surgical patients, 46 (66.7%) underwent curative resection and 23 (33.3%) underwent a palliative procedure, resulting in 67/69 (97.1%) surgical patients experiencing post-operative relief of their symptoms. The median survival period of the patients who underwent curative resection was 48.9 months compared with only 5.4 and 5.7 months in those undergoing palliative procedures and non-surgical interventions, respectively. Thus, surgery should be strongly considered as a treatment strategy for patients with gastric metastatic disease (21). In the present case, however, the patient refused to undergo gastrectomy and succumbed two months later.

In conclusion, to the best of our knowledge, the present study reports the first case of isolated gastric metastasis from a sphenoid sinus melanoma. The patient was firstly diagnosed with sphenoid sinus melanoma. After subtotal tumorectomy, the patient received radiotherapy, chemotherapy and immunotherapy. According to the RECIST guidelines (12), the patient remained in a stable disease state for 12 months. However, following gastroscopy and biopsy six months later, gastric metastasis was identified. Gastrectomy was recommended, however, the patient refused treatment and succumbed to the disease after two months. The present case demonstrated the importance of palliative surgery in patients with gastric metastasis as a result of melanoma.
